# Supplementary material for: Harnessing the potential of chloroplast-derived expression elements for enhanced production of cellulases in Escherichia coli
Source: PeerJ. 2025 Jan 31;13:e18616. doi: 10.7717/peerj.18616 (PMC11789652; doi:10.7717/peerj.18616)
Supplement: Supplemental Information 7 [file peerj-13-18616-s007.docx]

Table S1: List of primers used in this study for modifications of vector and genes

| **Primer** | **Sequence (5ʹ-3ʹ)** | **Purpose** |
| --- | --- | --- |
| AG122 | CG**CACCTGCAACGC**ATGGGTATATCTCCTTCTG | Forward primer to add PaqCI site in vector backbone. |
| AG123 | GG**CACCTGCGGAT**TAATCTAGAGAAATTCAATTAAGGAAATAAATT | Reverse primer to add PaqCI site in vector backbone. |
| AS133 | AG**CACCTGCTGCAC**ATGCATCACCATCACCAT | Forward primer to add PaqCI site in betaglucosidase gene. |
| AS134 | GC**CACCTGCGGAC**ATTAGTCTTCCAGACCGTT | Reverse primer to add PaqCI site in betaglucosidase gene. |
| AS136 | AG**CACCTGCTGCAC**ATGCATCATCATCATCATCAC | Forward primer to add PaqCI site in endoglucanase gene. |
| AS137 | GC**CACCTGCGGACA**TTACTCTCTAACTTCCAAATCTATAGAG | Reverse primer to add PaqCI site in endoglucanase gene. |

PaqCI site is shown in bold sequence
