# Supplementary material for: Harnessing the potential of chloroplast-derived expression elements for enhanced production of cellulases in Escherichia coli
Source: PeerJ. 2025 Jan 31;13:e18616. doi: 10.7717/peerj.18616 (PMC11789652; doi:10.7717/peerj.18616)
Supplement: Supplemental Information 8 — Ionic interactions in beta-glucosidases. Ionic interactions at certain positions are common among beta-glucosidases from thermophilic bacteria (T. maritima and T. neapolitana) and mesophilic organisms (Lactococcus lactis and Arabidopsis thaliana). However, at some other positions ionic interactions are only found in beta-glucosidases from thermophilic bacteria and are absent at structurally equivalent positions in beta-glucosidases from mesophilic organisms. The minimum distance (in Å) between side chain atoms of acidic and basic residues for establishing the ionic interaction is given in parentheses. [file peerj-13-18616-s008.docx]

**Table S4.** Ionic interactions in beta-glucosidases. Ionic interactions at certain positions are common among beta-glucosidases from thermophilic bacteria (*T. maritima* and *T. neapolitana*) and mesophilic organisms (*Lactococcus lactis* and *Arabidopsis thaliana*). However, at some other positions ionic interactions are only found in beta-glucosidases from thermophilic bacteria and are absent at structurally equivalent positions in beta-glucosidases from mesophilic organisms. The minimum distance (in Å) between side chain atoms of acidic and basic residues for establishing the ionic interaction is given in parentheses.

| **Thermophilic beta-glucosidases** | | | | **Mesophilic beta-glucosidases** | |
| --- | --- | --- | --- | --- | --- |
| ***T. maritima* [1UZ1]** | ***T. maritima* [2CBU]** | ***T. maritima* [2WBG]** | ***T. neapolitana***  **[5IDI]** | ***Lactococcus lactis* [1PBG]** | ***Arabidopsis thaliana* [7F3A]** |
| Arg77:Glu351 (3.07) | Arg77:Glu351 (3.06) | Arg77:Glu351 (3.01) | - | Arg72:Glu375 (2.81) | Arg92:Glu388 (2.95) |
| Arg84:Glu22 (2.85) | Arg84:Glu22 (2.86) | Arg84:Glu22 (2.79) | Arg82:Glu20 (2.80) | Arg79:Glu21 (2.48) | Arg99:Glu37 (2.81) |
| Arg371:Asp368 (2.74) | Arg371:Asp368 (2.69) | Arg371:Asp368 (2.73) | Arg369:Asp366 (2.83) | Arg394:Asp391 (3.27) | Arg410:Asp407 (2.74) |
| Arg426:Asp64 (2.89) | Arg426:Asp64 (2.88) | Arg426:Asp64 (2.78) | Arg424:Asp62 (2.76) | Arg449:Asp59 (2.89) | Arg466:Asp79 (2.87) |
| Lys429:Asp402 (2.74) | Lys429:Asp402 (2.72) | Lys429:Asp402 (2.68) | Lys427:Asp400 (2.73) | Lys452:Asp425 (2.65) | Lys469:Asp441 (2.72) |
| Arg209:Asp288 (2.87) | Arg209:Asp288 (2.83) | Arg209:Asp288 (2.86) | Arg207:Asp286 (2.95) | Lys204:Asp292 (3.08) | Arg228:Asp310 (2.95) |
| Arg315:Glu227 (3.33) | Arg315:Glu227 (3.18) | Arg315:Glu227 (3.07) | Arg313:Glu225 (2.97) | - | - |
| Lys336:Glu388 (2.52) | Lys336:Glu388 (2.48) | Lys336:Glu388 (2.83) | - | - | - |
| Arg157:Asp106 (3.01) | Arg157:Asp106 (2.83) | Arg157:Asp106 (2.81) | Arg155:Asp104 (2.75) | - | - |
| Lys159:Asp214 (2.94) | Lys159:Asp214 (2.82) | Lys159:Asp214 (2.74) | Lys157:Asp212 (3.00) | - | - |
| His367:Asp430 (2.70) | His367:Asp430 (2.73) | His367:Asp430 (2.64) | His365:Asp428 (2.63) | - | - |
