# Supplementary material for: Harnessing the potential of chloroplast-derived expression elements for enhanced production of cellulases in Escherichia coli
Source: PeerJ. 2025 Jan 31;13:e18616. doi: 10.7717/peerj.18616 (PMC11789652; doi:10.7717/peerj.18616)
Supplement: Supplemental Information 9 [file peerj-13-18616-s009.docx]

**Table S1. Secondary structure content (%) of endoglucanases**

| **Secondary structure element** | **Thermophilic endoglucanases** | | | | **Mesophilic endoglucanases** | |
| --- | --- | --- | --- | --- | --- | --- |
|  | ***T. maritima* [3AMH]** | ***T. maritima* [3AMM]** | ***Thermo-coccus sp. 2319x1* [7S8K]** | ***Pyrococcus furiosus* [3VGI]** | ***Streptomyces lividans* [2NLR]** | ***Streptomyces sp. 11AG8* [1OA4]** |
| **α-helix** | 4.7 | 4.7 | 4.4 | 4.1 | 4.5 | 5.0 |
| **β-sheet** | 60.9 | 59.6 | 55.3 | 56.7 | 56.8 | 56.8 |
| **Turn** | 12.5 | 12.9 | 12.8 | 15.6 | 12.2 | 12.2 |
| **Coil** | 21.9 | 22.7 | 27.5 | 23.7 | 26.6 | 26.1 |
