# Supplementary material for: Harnessing the potential of chloroplast-derived expression elements for enhanced production of cellulases in Escherichia coli
Source: PeerJ. 2025 Jan 31;13:e18616. doi: 10.7717/peerj.18616 (PMC11789652; doi:10.7717/peerj.18616)
Supplement: Supplemental Information 10 [file peerj-13-18616-s010.docx]

**Table S2.** Number of residues involved in the formation of outer β-sheets (Sheet A) in endoglucanases.

|  | **Thermophilic endoglucanases** | | | | **Mesophilic endoglucanases** | |
| --- | --- | --- | --- | --- | --- | --- |
|  | ***T. maritima* [3AMH]** | ***T. maritima* [3AMM]** | ***Thermo-coccus sp.* *2319x1* [7S8K]** | ***Pyrococcus furiosus* [3VGI]** | ***Streptomyces lividans* [2NLR]** | ***Streptomyces sp. 11AG8* [1OA4]** |
| **Sheet** | 50 | 48 | 54 | 50 | 21 | 36 |
