# Supplementary material for: Harnessing the potential of chloroplast-derived expression elements for enhanced production of cellulases in Escherichia coli
Source: PeerJ. 2025 Jan 31;13:e18616. doi: 10.7717/peerj.18616 (PMC11789652; doi:10.7717/peerj.18616)
Supplement: Supplemental Information 11 — Ionic interactions that are only found at certain positions in endoglucanases from thermophilic bacteria (Thermotoga maritima (3AMH and 3AMM), Thermococcus sp. 2319x1 (7S8K), and Pyrococcus furiosus (3VGI)) and absent at structurally equivalent positions in endoglucanases from mesophilic bacteria (Streptomyces lividans (2NLR), and Streptomyces sp. 11AG8 (1OA4)) are given. The minimum distance (in Å) between side chain atoms of acidic and basic residues for establishing the ionic interaction is given in parentheses. [file peerj-13-18616-s011.docx]

**Table S3.** Ionic interactions in endoglucanases. Ionic interactions that are only found at certain positions in endoglucanases from thermophilic bacteria (*Thermotoga maritima* (3AMH and 3AMM), *Thermococcus sp. 2319x1* (7S8K), and *Pyrococcus furiosus* (3VGI)) and absent at structurally equivalent positions in endoglucanases from mesophilic bacteria (*Streptomyces lividans* (2NLR), and *Streptomyces sp. 11AG8* (1OA4)) are given. The minimum distance (in Å) between side chain atoms of acidic and basic residues for establishing the ionic interaction is given in parentheses.

| **Thermophilic endoglucanases** | | | | **Mesophilic endoglucanases** | |
| --- | --- | --- | --- | --- | --- |
| **3AMH** | **3AMM** | **7S8K** | **3VGI** | **2NLR** | **1OA4** |
| Lys73:Glu67 (2.75) | Lys73:Glu67 (2.76) | Lys83:Glu77 (2.75) | Lys133:Glu127 (2.82) | - | - |
| Lys73:Glu22 (2.66) | Lys73:Glu22 (2.86) | Lys83:Glu30 (2.67) | Lys133:Glu80 (2.75) | - | - |
| Arg121:Asp132 (2.74) | Arg121:Asp132 (2.87) | - | Arg183:Asp194 (3.08) | - | - |
| Lys150:Glu153 (2.65) | Lys150:Glu153 (2.63) | Lys163:Glu166 (3.16) | Lys213:Glu216 (2.91) | - | - |
| Lys186:Glu153 (2.81) | Lys186:Glu153 (2.69) | Lys199:Glu166 (2.94) | Lys249:Glu216 (2.60) | - | - |
| Lys150:Glu170 (2.76) | Lys150:Glu170 (2.91) | Lys163:Glu183 (3.43) | Lys213:Glu233 (3.68) | - | - |
| Arg184:Glu170 (2.86) | Arg184:Glu170 (2.91) | Arg197:Glu183 (2.76) | Arg247:Glu233 (2.83) | - | - |
