# Supplementary figures and images for: Harnessing the potential of chloroplast-derived expression elements for enhanced production of cellulases in Escherichia coli
Source: PeerJ. 2025 Jan 31;13:e18616. doi: 10.7717/peerj.18616 (PMC11789652; doi:10.7717/peerj.18616)

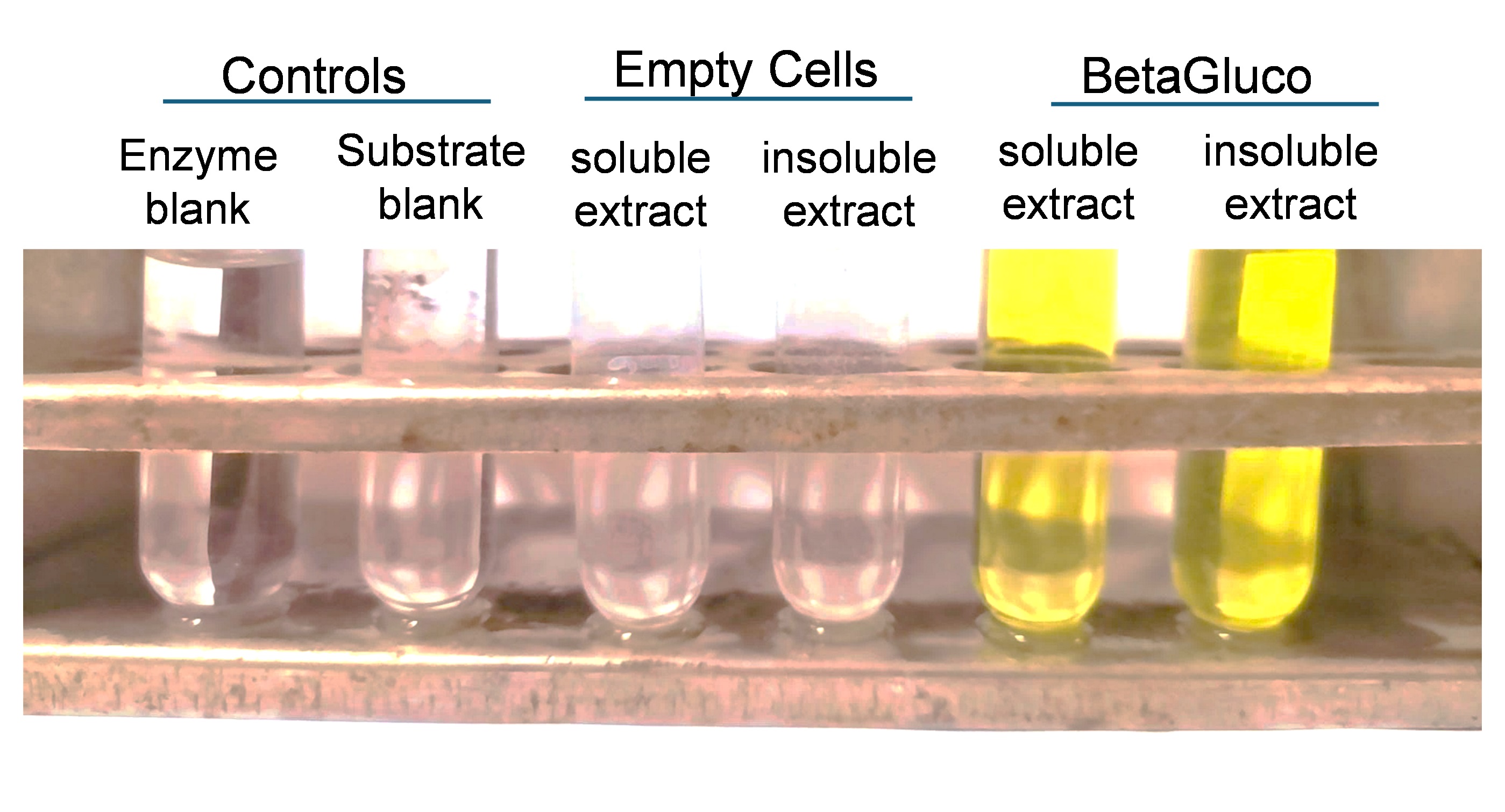

Supplement: Supplemental Information 12 — Pictures were taken after 30 min after initiating the assay. The experiment was repeated three times. Only representative pictures are shown. [file peerj-13-18616-s012.jpg]

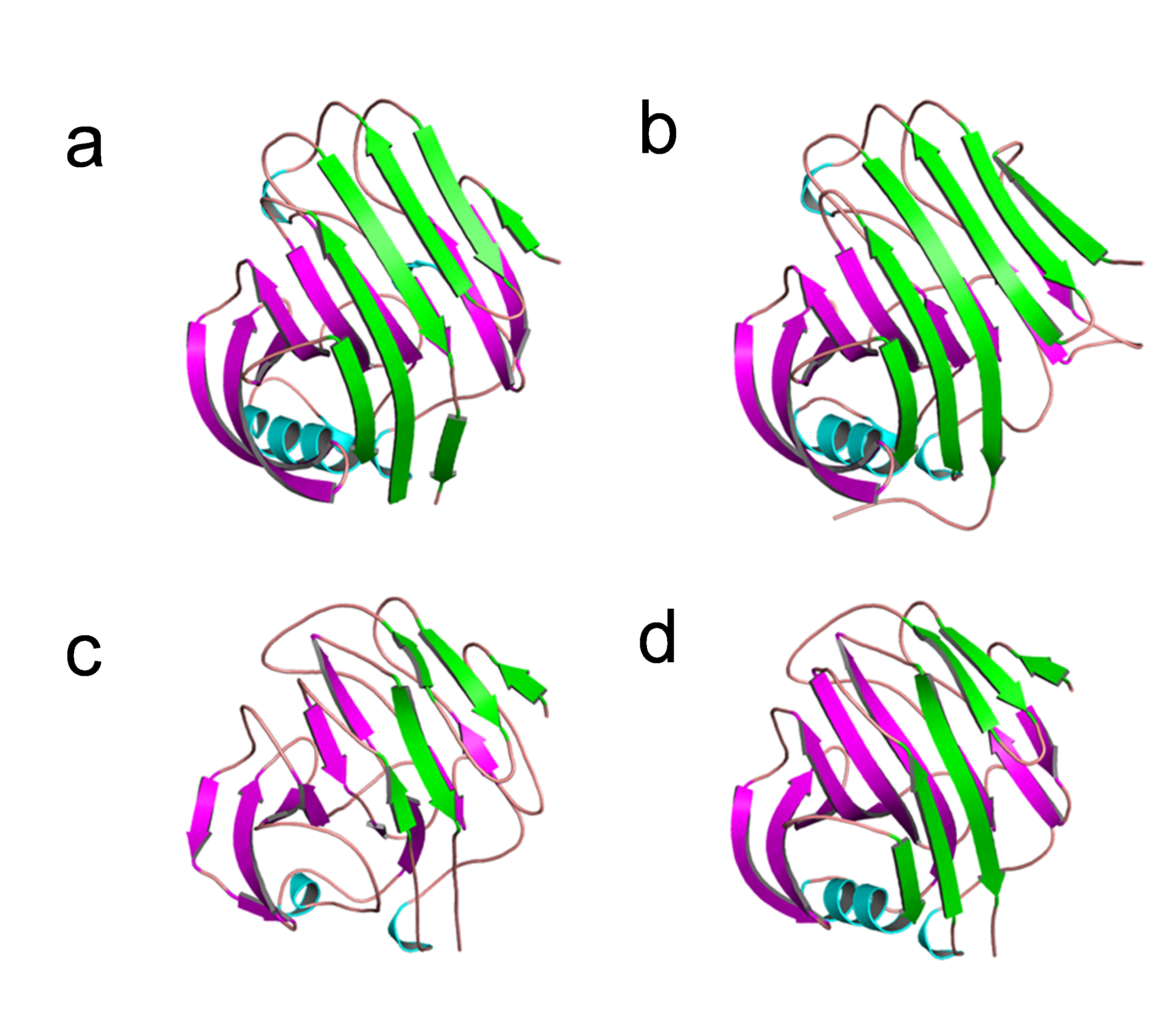

Supplement: Supplemental Information 13 [file peerj-13-18616-s013.png]
